# Cytotoxic indole alkaloids and polyketides produced by a marine-derived fungus *Aspergillus flavipes* DS720

**DOI:** 10.3389/fmicb.2022.959754

**Published:** 2022-07-22

**Authors:** An Xu, Xiang-Nan Xu, Mi Zhang, Chun-Lian Li, Li Liu, De-Yuan Fu

**Affiliations:** ^1^Clinical Medical College, Yangzhou University, Yangzhou, China; ^2^Department of Thyroid and Breast Surgery, Northern Jiangsu People's Hospital, Yangzhou, China; ^3^The School of Basic Medical Sciences, Fujian Medical University, Fuzhou, China; ^4^Department of General Surgery, Suqian First People's Hospital, Suqian, China

**Keywords:** indole alkaloids, polyketides, marine fungus, *Aspergillus flavipes*, cytotoxic activity

## Abstract

Marine-derived microorganisms possess the unique metabolic pathways to produce structurally novel secondary metabolites with potent biological activities. In this study, bioactivity-guided isolation of the marine deep-sea-derived fungus *Aspergillus flavipes* DS720 led to the characterization of four indole alkaloids (compounds **1**–**4**) and four polyketides (compounds **5**–**8**), such as two new indoles, flavonoids A (**1**) and B (**2**) with a C-6 reversed prenylation, and a new azaphilone, flaviazaphilone A (**5**). Their chemical structures were unambiguously established by an extensive interpretation of spectroscopic data, such as 1D/2D NMR and HRESIMS data. The absolute configurations of the new compound **5** were solved by comparing the experimental and calculated Electronic Circular Dichroism (ECD) spectra. Since sufficient amount of flavonoids A (**1**) was obtained, **1** was subjected to a large-scale cytotoxic activity screening against 20 different human tumor cell lines. The results revealed that **1** showed broad-spectrum cytotoxicities against HeLa, 5637, CAL-62, PATU8988T, A-375, and A-673 cell lines, with the inhibition rates of more than 90%. This study indicated that the newly discovered indole alkaloid **1** may possess certain potential for the development of lead compounds in the future.

## Introduction

Marine-derived microorganisms are widely distributed in the marine ecosystem. Marine microorganisms are subjected to various extreme environmental stresses, and therefore, they have evolved unique metabolic pathways to synthesize structurally novel secondary metabolites with potent biological activities (Jiang et al., 2020). Marine microorganisms are one of the most notable and prolific sources of bioactive natural products (Carroll et al., 2021). Although a large number of natural products have been discovered from marine microorganisms (Rateb and Ebel, 2011; Zhang et al., 2020), it is a matter of fact that, the trend toward finding new natural products is approaching saturation due to the redundancy of the isolation and characterization of microorganisms. Therefore, the discovery of new compounds from unexplored environments has proven to be an alternative strategy to search for microbial novelty. Extremophiles, which were isolated from the deep-sea, hydrothermal vents, cold water, and polar region, are largely unexplored (Soldatou and Baker, 2017). These microorganisms are extraordinarily adapted and metabolically active under extreme environmental conditions, which promote them to produce abundant novel secondary metabolites (Obulisamy and Mehariya, 2021).

**Figure 1:**
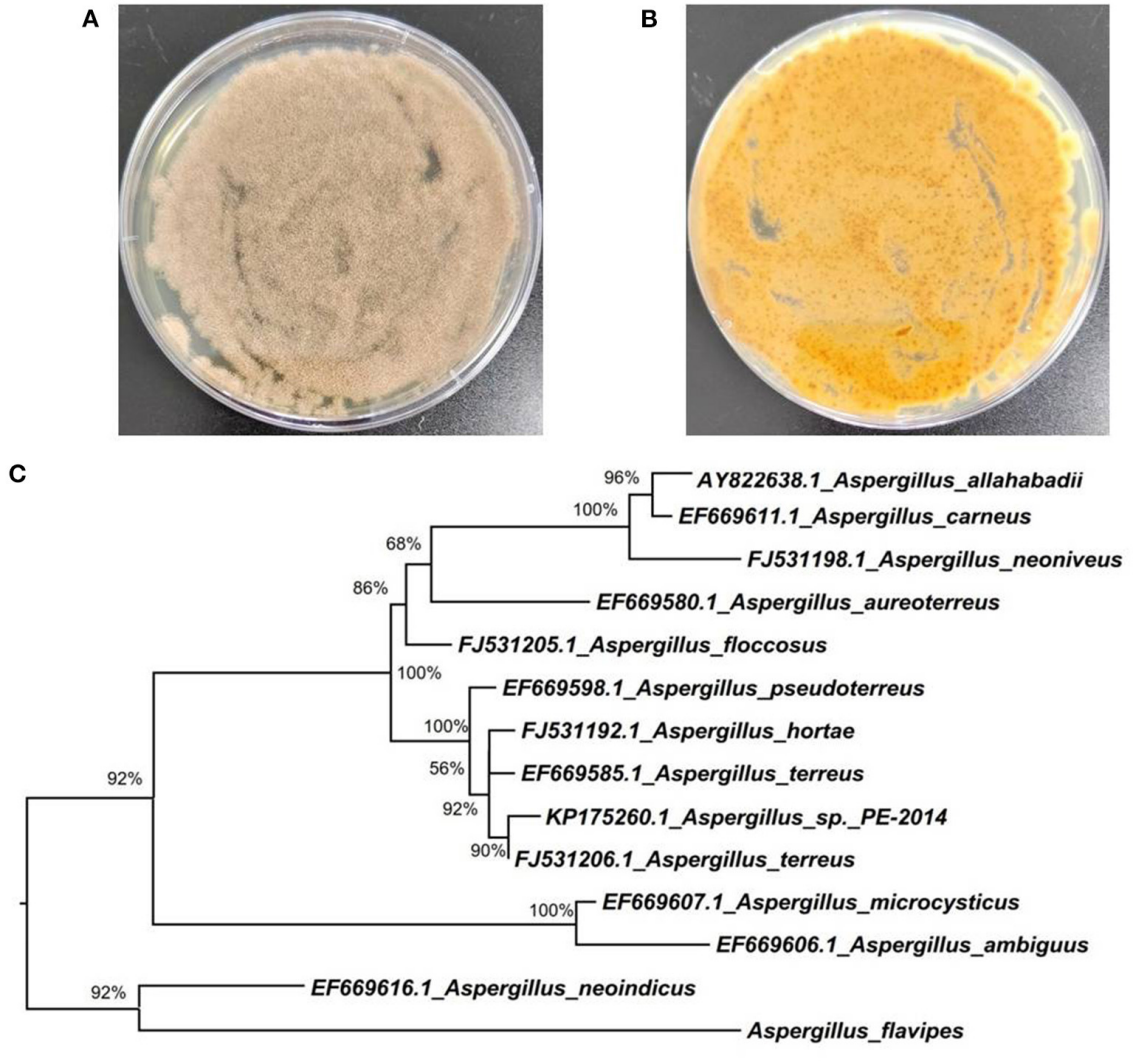
Morphology of *Aspergillus flavipes* DS720 cultured on PDA medium [**(A)**, front view; **(B)** reverse view]; **(C)** Neighbor-joining tree based on ITS and β-tubulin sequences.

**Figure 2:**
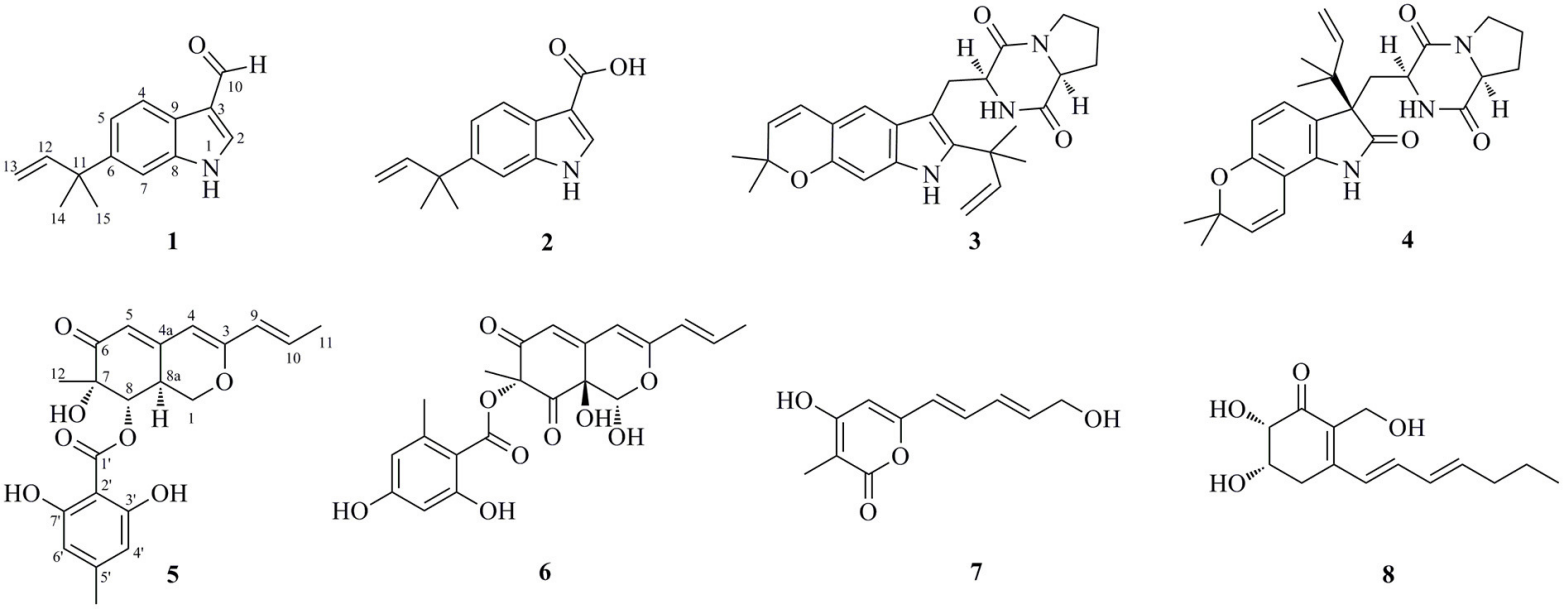
Chemical structures of the isolated compounds **1**–**8**.

Marine-derived fungi belonging to the genus *Aspergillus* have been widely studied for their biosynthetic potential for generating bioactive secondary metabolites, such as diverse polyketides (macrolides, phenols, quinines, and lactones), heterocyclic alkaloids, terpenoids, steroids, and other miscellaneous compounds (Xu et al., 2020). In our ongoing research on bioactive secondary metabolites from the deep-sea-derived fungi, an *Aspergillus flavipes* DS720 (Figure 1) was isolated from a deep seawater sample, which was collected from the Mariana Trench at a depth of 2,000 m. Preliminary cytotoxic screening indicated that the extracts of this fungal strain possessed considerable inhibitory effects on various human tumor cell lines. Especially, the extracts showed strong activities against HeLa, PATU8988T, A-375, and A-673 cell lines at the concentration of 40 mg/ml, with inhibition rates of 75, 82, 83, and 86%, respectively. Based on prescreening results, a large fermentation was performed. Subsequent chromatographic purification of the ethyl acetate extracts yielded eight compounds, such as four indole alkaloids (compounds **1**–**4**) and four polyketides (compounds **5**–**8**) (Figure 2). Among them, flavonoids A (**1**) and B (**2**) with a C-6 reversed prenylation, and an azaphilone, flavia azaphilone A (**5**), are new compounds. Since a sufficient amount of flavonoids A (**1**) was obtained (45 mg/20 g, pure compound/crude extract), a large-scale cytotoxic activity screening of **1** against 20 different human tumor cell lines was performed. Interestingly, **1** showed broad-spectrum cytotoxicities against HeLa, 5637, CAL-62, PATU8988T, A-375, and A-673 cell lines, with the inhibition rates of more than 90%. In this study, the isolation, structural elucidation, and cytotoxic activities of the new compound **1** are discussed herein.

## Materials and methods

### General

Specific rotation values were recorded on a JASCO P-1020 digital polarimeter (Tokyo, Japan). UV spectra were obtained with a Lambda 35 UV/Vis spectrophotometer (Perkin Elmer, Waltham, United States). Scientific LTQ Orbitrap XL spectrometer (Thermo Scientific, Waltham) was applied to measure the mass spectra of the new compounds. The 1D and 2D NMR spectra were measured with an Agilent DD2 spectrometer (Agilent Technologies, Santa Clara, United States, 500 MHz for ^1^H and 125 MHz for ^13^C). Chemical shifts (δ) were referenced to DMSO-*d*_6_ at 2.50 for ^1^H and 39.5 for ^13^C. Open column chromatography (CC) was performed by silica gel (200–300 mesh, Qingdao Marine Chemical Factory, Qingdao, China), octadecylsilyl (ODS) reversed-phase gel (30–50 μm, YMC CO., LTD., Japan), and Sephadex LH-20 (GE Healthcare, United States). All solvents used were of either analytical grade or filtered prior to use.

### Fungal material

The fungal strain DS720 used in this study was isolated from a deep seawater sample, which was collected from the Mariana Trench at a depth of 2,000 m (N 11°21.738′, E 142°29.307′). This fungus was preliminarily identified as *Aspergillus flavipes* by a standard molecular biological protocol. The sequence analysis of the internal transcribed spacer (ITS) region of the rDNA shared a 99% match to *A. flavipes* NRRL 5175 (Accession No. EF661428) in the BLASTn search. Then, sequences of the ITS (GenBank Accession No. ON340751) and β-tubulin genes from *Aspergillus* species were aligned by MEGA version 6.0 and manually improved when necessary. Subsequently, the phylogenetic tree of the combined dataset was made on the basis of maximum-likelihood (ML) analysis with MEGA version 6.0 with 1,000-generation bootstrap values, for which a value ≥ 50% was considered significantly (Figure 1). A voucher strain of this fungus was deposited at School of Life Sciences, Nanjing University.

### Cultivation and extraction

The cultivation of the fungal strain DS720 was performed in 1 L Erlenmeyer flasks containing commercially available PDB medium (potato dextrose broth, Solarbio Life Sciences CO., LTD., Beijing, China). The mycelium from each culture plate was inoculated in a 500 ml Erlenmeyer flask, which was filled with 200 ml of PDB medium supplemented with 3% sea salt. Then, the flask culture was subjected to a rotary shaker at 200 rpm as seed cultures. Following cultivation for 5 days, the seed cultures were transferred into autoclaved 1 L Erlenmeyer flasks with PDB medium. The fermentation process was carried out under static conditions and daylight for 30 days. After the fermentation, the cultures (~30 L) were filtered to separate the broth and mycelia layer. The broth was extracted adequately with EtOAc for three times, while the mycelia were crushed and extracted with EtOAc. The combined EtOAc extracts were evaporated under reduced pressure to yield 20 g of a crude gum.

### Isolation and purification

The obtained crude gum was subjected to open silica gel CC with a stepwise mixed CH_2_Cl_2_/MeOH solvent system with the ratios of 100:1, 50:1, 25:1, 10:1, 5:1, and 1:1 (v/v) to yield six fractions (Fr.1–6). Fr.2, eluted with CH_2_Cl_2_/MeOH 50:1, was fractionated by ODS reversed-phase gel column with a stepwise solvent system of MeOH/H_2_O (from 20 to 90%) to give subfractions Fr.2.1–2.6. The Fr.2.2 was purified by semi-preparative HPLC eluting with 50% MeOH–H_2_O to obtain compound **3** (*t*_R_ 12.6 min; 2.1 mg), while Fr.2.4 was applied to a Sephadex LH-20 (MeOH) to give compound **4** (1.8 mg). Fr.3, eluted with CH_2_Cl_2_/MeOH 25:1, was afforded to silica gel CC (CH_2_Cl_2_/MeOH, from 30:1 to 5:1) to give two subfractions, Fr.3.1 and Fr.3.2. Compound **1** (*t*_R_ 16.0 min; 45 mg) was isolated from Fr.3.1 as the main ingredient components by semi-preparative HPLC (60% MeOH–H_2_O). Purification of Fr.3.2 by semi-preparative HPLC using 55% MeOH–H_2_O obtained compound **2** (*t*_R_ 10.5 min; 3.2 mg). Fr.4 (eluted with CH_2_Cl_2_/MeOH 10:1) and Fr.5 (eluted with CH_2_Cl_2_/MeOH 5:1) were combined and then fractionated with silica gel CC (CH_2_Cl_2_/MeOH, from 20:1 to 1:1) to obtain three subfractions Fr.4.1–4.3. Compound **7** (2.6 mg) was isolated from Fr.4.1 by preparative TLC (pTLC) eluting with MeOH/H_2_O 20:1, whereas compound **8** (2.0 mg) was obtained from Fr.4.2 by pTLC eluting with CH_2_Cl_2_/MeOH/acetic acid 20:1:0.5. Fr.4.3 was subjected to semi-preparative HPLC (55% MeOH–H_2_O) to give compounds **5** (*t*_R_ 10.0 min; 1.9 mg) and **6** (*t*_R_ 15.2 min; 1.2 mg).

Flavonoid A [**1**, 6-(2-methylbut-3-en-2-yl)-1*H*-indole-3-carbaldehyde]: colorless oil; UV (MeOH) λ_max_ (log ε) 204 (4.28), 229 (4.24), 292 (3.54) nm; ^1^H and ^13^C NMR data, see Table 1; HRESIMS *m/z* 214.1223 [M + H]^+^ (calcd for C_14_H_16_NO, 214.1226); *m/z* 236.1044 [M + Na]^+^ (calcd for C_14_H_15_NONa, 236.1046).

**Table 1:** ^**1**^H NMR (500 MHz, **δ** in ppm) and ^**13**^C NMR Data (125 MHz, **δ** in ppm) for **1** and **2** (measured in DMSO-*d*_**6**_).

**Postion**	**Compound 1**		**Compound 2**	
	δ_H_ **(*****J*** **in Hz)**	δ_C_**, type**	δ_H_ **(*****J*** **in Hz)**	δ_C_**, type**
1-NH	11.25, br s	–	10.84, br s	–
2	8.17, s	138.1, CH	7.83, d (2.1)	131.6, CH
3		117.8, C		107.5, C
4	7.18, d (8.5)	120.5, CH	7.09, s	119.4, CH
5	8.05, dd (8.5, 1.8)	119.5, CH	7.96, dd (8.4, 1.9)	119.1, CH
6		132.1, C		131.7, C
7	7.18, br s (overlapped)	122.2, CH	7.10, s	120.9, CH
8		134.2, C		133.5, C
9		125.2, C		127.1, C
10	9.95, s	185.0, CH		166.1, C
11		40.2, C		40.2, C
12	6.15, dd (17.5, 10.6)	146.3, CH	6.12, dd (17.5, 10.6)	146.4, CH
13	5.08, d (10.6) 4.98, d (17.5)	112.4, CH_2_	5.07, d (10.6) 5.00, d (17.5)	112.2, CH_2_
14	1.50, s	27.5, CH_3_	1.48, s	27.4, CH_3_
15	1.50, s	27.5, CH_3_	1.48, s	27.4, CH_3_

Flavonoid B [**2**, 6-(2-methylbut-3-en-2-yl)-1*H*-indole-3-carboxylic acid]: colorless oil; UV (MeOH) λ_max_ (log ε) 203 (4.32), 231 (4.22), 296 (3.49) nm; ^1^H and ^13^C NMR data, see Table 1; HRESIMS *m/z* 230.1179 [M + H]^+^ (calcd for C_14_H_16_NO_2_, 230.1176).

Flaviazaphilone A [**5**, (7*S*,8*S*,8a*S*)-7-hydroxy-7-methyl-6-oxo-3-((*E*)-prop-1-en-1-yl)-6,7,8,8a-tetrahydro-1*H*-isochromen-8-yl 2,6-dihydroxy-4-methylbenzoate]: colorless oil; ${\left[\alpha \right]}_{\text{D}}^{20}$ + 72.5 (*c* 0.05, MeOH); UV (MeOH) λ_max_ (log ε) 211 (4.36), 268 (3.96), 317 (3.85), 340 (3.84) nm; ^1^H and ^13^C NMR data, see Table 2; HRESIMS *m/z* 387.1433 [M + H]^+^ (calcd for C_21_H_23_O_7_, 387.1438).

**Table 2:** ^**1**^H NMR (500 MHz, **δ** in ppm) and ^**13**^C NMR Data (125 MHz, **δ** in ppm) for **5** (measured in DMSO-*d*_**6**_).

**Postion**	δ_H_ **(*****J*** **in Hz)**	δ_C_**, type**
1	4.62, dd (10.6, 5.2)	68.0, CH_2_
3		159.7, C
4	5.74, s	103.4, CH
4a		150.8, C
5	5.68, d (1.5)	116.7, CH
6		195.1, C
7		73.8, C
8	5.02, d (10.0)	75.5, CH
8a	3.18, m	35.1, CH
9	6.00, d (15.9)	126.0, CH
10	6.29, m	133.4, CH
11	1.79, d (7.4)	18.6, CH_3_
12	1.10, s	19.8, CH_3_
1'		169.2, C
2'		109.9, C
3'		161.1, C
4'	6.16, d (2.3)	100.9, CH
5'		140.1, C
6'	6.14 d (2.3)	110.1, CH
7'		159.6, C
8'	2.25, s	21.8, CH_3_

### Computational section

The conformer rotamer ensemble sampling tool (crest) was utilized to generate candidate conformers and DFT calculations were performed using the Gaussian 16 program (Frisch et al., 2016). The conformers within an energy window of 10 kcal/mol were optimized at B3LYP/6-31G(d) level of theory with Grimme's D3 dispersion correction (“EmpiricalDispersion = GD3” key words in input files). Frequency analysis of all optimized conformations was undertaken at the same level of theory to ensure they were true local minima on the potential energy surface. Then, energies of all optimized conformations were evaluated by M062X/6-311+G(2d,p) with D3 dispersion correction. Gibbs free energies of each conformer were calculated by adding “Thermal correction to Gibbs Free Energy” obtained by frequency analysis to electronic energies obtained at M062X/6-311+G(2d,p). Room-temperature (298.15 K) equilibrium populations were calculated according to the Boltzmann distribution law. Those conformers accounting for over 2% population were subjected to subsequent calculations. Time-dependent density-functional theory (TDDFT) ECD calculations were run at Cam-B3LYP/TZVP level of theory in MeOH with IEFPCM solvent model, respectively. For each conformer, 30 excited states were calculated. The calculated ECD curves were generated using Multiwfn 3.6 software.

### Cytotoxic assay

Cytotoxic activities of the crude exacts and the new compound **1** against 20 different human tumor cells (human lung cancer cells A549, human breast cancer cells MCF7, human gastric carcinoma cells MKN-45, human colon cancer cells HCT 116, human hepatoma cell lines HepG2, human cervical carcinoma cells HeLa, human chronic myelogenous leukemia cells K-562, human brain tumor stem cells SF126, human ovarian teratoma cells PA-1, human renal clear cell adenocarcinoma cells 786-O, human esophageal cancer cells TE-1, human bladder cancer cells 5,637, human prostatic cancer cells DU 145, human thyroid cancer cells CAL-62, human pancreatic cancer cells PATU8988T, human osteosarcoma cells HOS, human malignant melanoma cells A-375, human rhabdomyosarcoma cells A-673, human pharyngeal squamous cells FaDu, and human gallbladder carcinoma cells GBC-SD) were evaluated by the CCK-8 method (Chen et al., 2021). The tested cells were treated with 40 mg/ml of compound samples. A total of 10 μl of 5 g/L CCK-8 solutions were added to each well at 48 h. The cell lines were then incubated at 37°C for 1.5 h. Absorbance data were obtained with a microplate spectrophotometer reader at 490 nm. Commercial doxorubicin was used as the positive control.

### Statistical analysis

The data were statistically analyzed using SPSS software (Version 18.0, Chicago, IL, USA) and were expressed as the means ± *SD*.

## Results and discussion

### Structural elucidation of the new compounds

**Figure 3:**
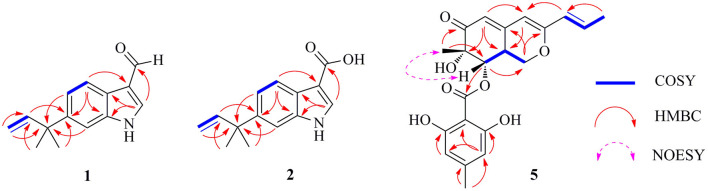
Selected Homonuclear chemical shift Correlation Spectroscopy (COSY), eteronuclear Multiple-Bond Correlation (HMBC), and Nuclear Overhauser Effect Spectroscopy (NOESY) correlations of **1**, **2**, and **5**.

Flavonoid A (**1**) was obtained as colorless oil. The molecular formula of **1** was deduced as C_14_H_15_NO by the observation of [M + H]^+^ and [M + Na]^+^ ion peaks in the HRESIMS spectrum at *m/z* 214.1223 and 236.1044, respectively. The ^1^H NMR spectroscopic data (Table 1) exhibited the presence of one isolated NH signal at δ_H_ 11.25 (1H, br s, 1-NH), two overlapped methyl groups at δ_H_ 1.50 (6H, s, H_3_-14, and H_3_-15), a set of terminal methylene signal at δ_H_ 5.08 (1H, d, *J* = 10.6 Hz, H-13α) and 4.98 (1H, d, *J* = 17.5 Hz, H-13β), six methines such as three aromatic at δ_H_ 7.18 (1H, d, *J* = 8.5 Hz, H-4), 8.05 (1H, dd, *J* = 8.5, 1.8 Hz, H-5), and 7.18 (1H, br s, H-7), two olefinic at δ_H_ 8.17 (1H, s, H-2) and 6.15 (1H, dd, *J* = 17.5, 10.6 Hz, H-12), and one aldehyde group at δ_H_ 9.95 (1H, s, H-10). The ^13^C NMR combined with DEPT spectra revealed 14 carbon signals, which were classified into one aldehyde carbon at δ_C_ 185.0 (C-10), one terminal methylene group at δ_C_ 112.4 (C-13), five aromatic/olefinic methines, five quaternary carbons, and two methyl groups. Eight characteristic aromatic/olefinic carbons (C-2–C-9) along with the NH signal (1-NH) hinted at the presence of an indole moiety containing a 1,2,5-trisubstituted benzene ring. The key eteronuclear Multiple-Bond Correlation (HMBC) correlations from H_2_-13 to C-11 and C-12, from H-12 to C-6, and from H_3_-14 to C-12 revealed a *trans*-isopentene group. The definite HMBC correlations from the two *gem*-methyl groups of the isopentene (H_3_-14 and H_3_-15) to C-6 as well as correlations from H-5 and H-7 to C-11 indicated that the prenylation was occurred at C-6 of the benzene (Figure 3). Moreover, the aldehyde group was attached to C-3 as evidenced from the HMBC correlation from H-2 to C-10 (Figure 3). Accordingly, the structure of compound **1** was established.

Flavonoid B (**2**) was also obtained as colorless oil and its molecular formula was determined by HRESIMS data. The HRESIMS spectrum of **2** showed a prominent pseudomolecular ion peak at *m/z* 230.1179 [M + H]^+^, which was attributed to the molecular formula of C_14_H_15_NO_2_. With compound **1** in hand, the structural elucidation of **2** was straightforward. Investigation of the 1D NMR data (Table 1) confirmed that **2** possessed high structural similarity with **1**. Compared with the ^1^H and ^13^C NMR data of **1**, the main difference was that **2** had an additional ester carbonyl at δ_C_ 166.1 (C-10), rather than the aldehyde group in **1**. In addition, the chemical shifts at C-2 (δ_C_ 131.6) and C-3 (δ_C_ 107.5) in **2** were changed significantly. The ester carbonyl group was deduced to be located at C-2 based on the HMBC correlation from H-2 to C-10 (Figure 3). With the aid of detailed analysis of 1D and 2D NMR data, compound **2** was characterized as a new indole with a C-6 reversed prenylation.

Flaviazaphilone A (**5**) was isolated as colorless oil. The HRESIMS of **5** gave a molecular formula of C_21_H_22_O_7_ (*m/z* 387.1433 [M + H]^+^, calcd for C_21_H_23_O_7_, 387.1438). The NMR data of **5** (Table 2) indicated the presence of one ketone carbonyl at δ_C_ 195.1 (C-6), one ester carbonyl at δ_C_ 169.2 (C-1'), seven quaternary carbons such as six sp^2^ and one oxygenated sp^3^ at δ_C_ 73.8 (C-7), six aromatic/olefinic methines at δ_C_ 133.4 (C-10), 126.0 (C-9), 116.7 (C-5), 110.1 (C-6'), 103.4 (C-4), and 100.9 (C-4'), two sp^3^ methines such as one oxymethine at δ_C_ 75.5 (C-8), one oxygenated methylene at δ_C_ 68.0 (C-1), and three methyl groups at δ_C_ 21.8 (C-8'), 19.8 (C-12), and 18.6 (C-11). The ^1^H–^1^H COSY correlations indicated the presence of two spin systems, CHO–CH–CH_2_O and CH=CH–CH_3_ (Figure 3). The mutual HMBC correlations shown in Figure 3 revealed that **5** possessed an azaphilone skeleton. These spectroscopic features suggested the presence of a similar azaphilone skeleton with that of berkazaphilone C, which was previously isolated from an extremophilic fungus *Penicillium rubrum* (Stierle et al., 2012). The main differences were the substituents of the benzene ring. Key HMBC correlations from H-8' to C-4' and C-5', from H-6' to C-7', from H-4' to C-2' and C-3' constructed the substructure of 1,2,3,5-tetrasubstituted benzene ring.

**Figure 4:**
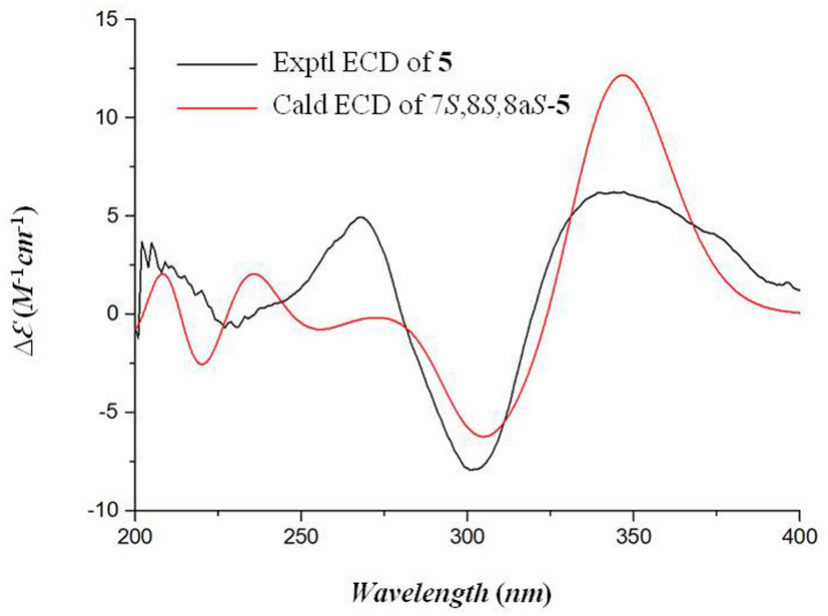
The experimental and calculated ECD spectra of compound **5**.

The relative stereochemistry of **5** was established by interpretation of NOESY spectrum and ^3^*J*-coupling data. The NOE correlation between H_3_-12 and H-8 indicated a cofacial relationship between H_3_-12 and H-8 (Figure 3). In addition, ^3^*J*-coupling data showed *ax*/*ax* interactions between H-8 and H-8a (*J* = 10.0 Hz) (Stierle et al., 2012). Furthermore, the large coupling constant between H-9 and H-10 (*J* = 15.9 Hz) illustrated that the double bond at C-9 and C-10 was in the *E* configuration. To determine the absolute configurations of **5**, ECD computations for B3LYP/6-311+G(d)-optimized conformers were carried out at Cam-B3LYP/TZVP level. The experimental and calculated ECD spectra of **5** exhibited high consistency (Figure 4), and thus finally determined the absolute configurations of **5** as 7*S*, 8*S*, 8a*S*.

In addition to the new compounds **1**, **2**, and **5**, five related known compounds (**3**, **4**, **6**–**8**) were obtained from this fungal strain. Based on comparison of their spectroscopic data with those in the literatures, they were identified as dihydrocarneamide A (**3**) (Zhang et al., 2015), notoamide C (**4**) (Kato et al., 2007), purpurquinone A (**6**) (Wang et al., 2011), saturnispol G (**7**) (Meng et al., 2018), and palitantin B (**8**) (Yang et al., 2020), respectively.

### Cytotoxic activity

**Figure 5:**
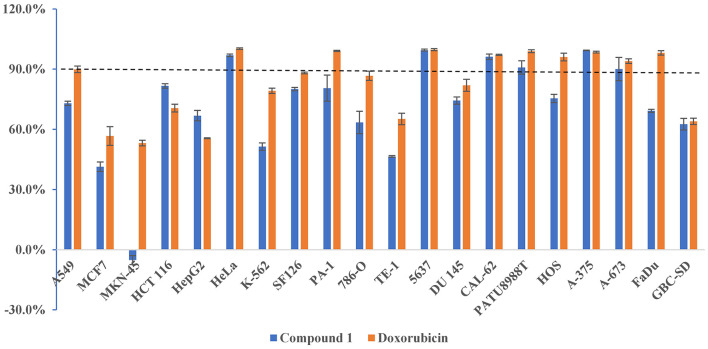
Cytotoxicities of compound **1** against 20 different human tumor cell lines.

The new compounds **1**, **2**, and **5** were evaluated to determine their cytotoxic activity against HeLa cell lines. Compound **1** exhibited obvious cytotoxicity with the inhibition rate of (96.94 ± 0.62) % at the concentration of 20 μM. Since sufficient amount of **1** was obtained (45 mg/20 g, pure compound/crude extract), a large-scale cytotoxic activity screening of **1** against 20 different human tumor cell lines was performed. As a result, **1** showed high and broad-spectrum cytotoxicities against HeLa, 5637, CAL-62, PATU8988T, A-375, and A-673 cell lines, with the inhibition rates of (96.94 ± 0.62) %, (99.49 ± 0.50) %, (96.16 ± 1.34) %, (90.83 ± 3.31) %, (99.32 ± 0.11) %, and (90.01 ± 5.81) %, respectively (Figure 5). In particularly, since thyroid cancer is one of the leading cancers worldwide, chemotherapy is currently needed. Compound **1** showed strong activity against human thyroid cancer cells CAL-62 (96.16%), with an IC_50_ value of 10.4 μM, indicating that it may possess certain potential for the development of antitumor lead compounds. Moreover, further studies should focus on cytotoxicity assay on normal cell lines to check the specificity of the cytotoxicity. The safety index for cytotoxicity assay will reveal the true cytotoxic potential of the isolated compounds.

## Conclusions

In conclusion, four indole alkaloids (compounds **1**–**4**) and four polyketides (compounds **5**–**8**) were isolated and identified from the deep-sea-derived fungus *Aspergillus flavipes* DS720. Among them, the indoles flavonoids A (**1**) and B (**2**) and the azaphilone flaviazaphilone A (**5**) are new compounds. Flavonoids A (**1**) and B (**2**) represent rare examples with a C-6 reversed prenylation. The structures of the new compounds were determined by analysis of NMR data, HRESIMS, and TDDFT ECD calculations. In the screening of cytotoxicities of **1** against 20 different human tumor cell lines, **1** showed high and broad-spectrum cytotoxicity against HeLa, 5637, CAL-62, PATU8988T, A-375, and A-673 cell lines. This study indicated that the deep-sea-derived microbes were considered to be valuable resources for the development of new drugs. Meanwhile, the newly discovered indole alkaloid **1** may be a promising antitumor lead compound.

## Data availability statement

The original contributions presented in the study are included in the article/supplementary material, further inquiries can be directed to the corresponding author.

## Author contributions

AX and X-NX: conceptualization and writing—original draft preparation. AX, X-NX, MZ, and C-LL: experiment implementation. LL: data processing. D-YF: writing—review and editing. All authors have read and approved the final manuscript.

## Funding

This work was supported by the National Natural Science Foundation of China (82072909).

## Conflict of interest

The authors declare that the research was conducted in the absence of any commercial or financial relationships that could be construed as a potential conflict of interest.

## Publisher's note

All claims expressed in this article are solely those of the authors and do not necessarily represent those of their affiliated organizations, or those of the publisher, the editors and the reviewers. Any product that may be evaluated in this article, or claim that may be made by its manufacturer, is not guaranteed or endorsed by the publisher.
