# Supplementary material for: Cytotoxic indole alkaloids and polyketides produced by a marine-derived fungus Aspergillus flavipes DS720
Source: Front Microbiol. 2022 Jul 22;13:959754. doi: 10.3389/fmicb.2022.959754 (PMC9355579; doi:10.3389/fmicb.2022.959754)
Supplement: Supplementary file 1 [file Data_Sheet_1.docx]

***Supplementary Material***

Cytotoxic Indole Alkaloids and Polyketides Produced by a Marine-Derived Fungus *Aspergillus flavipes* DS720

An Xu^1†^, Xiang-Nan Xu^2†^, Mi Zhang^3^, Chun-Lian Li^2^, Li Liu^4^, De-Yuan Fu^2^*

^1^Medical College of Yangzhou University, Yangzhou 225009, Jiangsu, People’s Republic of China

^2^Department of Thyroid and Breast Surgery, Northern Jiangsu People’s Hospital, Yangzhou 225001, Jiangsu, People’s Republic of China

^3^The School of Basic Medical Sciences, Fujian Medical University, No. 1, Xuefu North Road, Fuzhou 350122, Fujian, People’s Republic of China

^4^Department of General Surgery, Suqian First People's Hospital, Suqian 223800, Jiangsu, People’s Republic of China

***Correspondence:**

De-Yuan Fu

fdy1003@163.com

^†^These authors contributed equally to this work.

Keywords: Indole alkaloids, Polyketides, Marine fungus, *Aspergillus flavipes*, Cytotoxic activity

**Contents**

**Table S1.** Conformational analysis of the B3LYP/6-31G(d) optimized conformers of **5** in the gas phase (T=298.15 K)

**Table S2.** Key transitions, oscillator strengths, and rotatory strengths in the ECD spectrum of conformer **5-1** at the Cam-B3LYP/TZVP level of theory in MeOH with IEFPCM solvent model.

**Table S3.** Key transitions, oscillator strengths, and rotatory strengths in the ECD spectrum of conformer **5-2** at the Cam-B3LYP/TZVP level of theory in MeOH with IEFPCM solvent model.

**Figure S1.** ^1^H NMR (500 MHz, DMSO-*d*_6_) spectrum of compound **1**

**Figure S2.** ^13^C NMR (125 MHz, DMSO-*d*_6_) spectrum of compound **1**

**Figure S3.** HSQC spectrum of compound **1**

**Figure S4.** COSY spectrum of compound **1**

**Figure S5.** HMBC spectrum of compound **1**

**Figure S6.** HRESIMS spectrum of compound **1**

**Figure S7.** ^1^H NMR (500 MHz, DMSO-*d*_6_) spectrum of compound **2**

**Figure S8.** ^13^C NMR (125 MHz, DMSO-*d*_6_) spectrum of compound **2**

**Figure S9.** HSQC spectrum of compound **2**

**Figure S10.** COSY spectrum of compound **2**

**Figure S11.** HMBC spectrum of compound **2**

**Figure S12.** HRESIMS spectrum of compound **2**

**Figure S13.** ^1^H NMR (500 MHz, DMSO-*d*_6_) spectrum of compound **5**

**Figure S14.** ^13^C NMR (125 MHz, DMSO-*d*_6_) spectrum of compound **5**

**Figure S15.** HSQC spectrum of compound **5**

**Figure S16.** COSY spectrum of compound **5**

**Figure S17.** HMBC spectrum of compound **5**

**Figure S18.** NOESY spectrum of compound **5**

**Figure S19.** HRESIMS spectrum of compound **5**

**Figure S20.** Chiral HPLC analysis of compound **5** (Whelk-O1 rpirkle-cencept chiral HPLC column, *n*-hexane−ethanol 10:1, 1.0 mL/min)

**Table S1.** Conformational analysis of the B3LYP/6-31G(d) optimized conformers of **5** in the gas phase (T=298.15 K)

| **Conformer** | **E*^a^* (Hartree)** | **C*^b^* (Hartree)** | **G*^c^* (kcal/mol)** | **ΔG*^d^* (kcal/mol)** | **Population*^e^*** |
| --- | --- | --- | --- | --- | --- |
| **5-1** | -1339.944391 | 0.335484 | -840604.5893 | 0 | 58.83% |
| **5-2** | -1339.944381 | 0.335811 | -840604.3779 | 0.2113922 | 41.17% |

**Table S2.** Key transitions, oscillator strengths, and rotatory strengths in the ECD spectrum of conformer **5-1** at the Cam-B3LYP/TZVP level of theory in MeOH with IEFPCM solvent model.

| ***Num^a^*** | ***Transition^b^*** | ***CI-coeff^b^*** | ***ΔE (eV)^d^*** | ***λ (nm)^e^*** | ***f^f^*** | ***R_vel_^g^*** | ***R_len_^h^*** |
| --- | --- | --- | --- | --- | --- | --- | --- |
| 1 | 102->103 | 0.69614 | 3.6732 | 337.54 | 1.0811 | 127.9193 | 129.1884 |
| 2 | 99->103 | 0.62204 | 3.9065 | 317.38 | 0.0060 | -38.2523 | -39.204 |
|  | 99->105 | 0.26866 |  |  |  |  |  |
| 3 | 101->104 | 0.68186 | 4.2058 | 294.80 | 0.1031 | -49.2388 | -50.8202 |
| 4 | 100->104 | 0.66995 | 5.0464 | 245.69 | 0.4721 | -11.8255 | -11.1131 |
| 5 | 101->103 | 0.69938 | 5.1377 | 241.32 | 0.0006 | -0.919 | -0.8756 |
| 6 | 102->104 | 0.69464 | 5.4160 | 228.92 | 0.0054 | -1.7459 | -1.9725 |
| 7 | 97->103 | -0.23706 | 5.4652 | 226.86 | 0.1568 | 16.2474 | 16.5611 |
|  | 98->103 | 0.6223 |  |  |  |  |  |
| 8 | 95->104 | 0.48097 | 5.7281 | 216.45 | 0.0001 | -0.1988 | 0.0401 |
|  | 96->104 | 0.37892 |  |  |  |  |  |
| 9 | 100->103 | 0.65491 | 5.8056 | 213.56 | 0.0018 | -2.4518 | -2.4555 |
| 10 | 97->103 | 0.59318 | 5.8426 | 212.21 | 0.0124 | -1.5688 | -1.6847 |
|  | 98->103 | 0.24387 |  |  |  |  |  |
| 11 | 102->105 | 0.65979 | 5.9233 | 209.32 | 0.0896 | -6.6128 | -5.3017 |
| 12 | 101->106 | 0.66326 | 6.0811 | 203.88 | 0.5853 | 19.755 | 20.593 |
| 13 | 95->103 | -0.28755 | 6.1113 | 202.88 | 0.0082 | -16.498 | -17.8642 |
|  | 96->103 | 0.50021 |  |  |  |  |  |
| 14 | 92->103 | 0.47776 | 6.3349 | 195.72 | 0.0037 | 5.9353 | 5.924 |
|  | 93->103 | 0.45242 |  |  |  |  |  |
| 15 | 100->106 | 0.59317 | 6.4226 | 193.04 | 0.1034 | -0.2435 | -0.1925 |
|  | 101->108 | 0.28383 |  |  |  |  |  |
| 16 | 93->103 | -0.2324 | 6.6037 | 187.75 | 0.0074 | -15.1081 | -15.6069 |
|  | 99->104 | 0.55101 |  |  |  |  |  |
| 17 | 92->103 | -0.30742 | 6.6550 | 186.30 | 0.0038 | -3.7308 | -3.4899 |
|  | 93->103 | 0.3535 |  |  |  |  |  |
|  | 99->104 | 0.39792 |  |  |  |  |  |
| 18 | 99->103 | -0.25627 | 6.8438 | 181.16 | 0.0017 | 9.3151 | 9.288 |
|  | 99->105 | 0.45105 |  |  |  |  |  |
| 19 | 102->107 | -0.27779 | 6.9098 | 179.43 | 0.0021 | -9.3708 | -8.0457 |
|  | 102->108 | -0.26559 |  |  |  |  |  |
|  | 102->109 | 0.4322 |  |  |  |  |  |
|  | 102->113 | 0.28713 |  |  |  |  |  |
| 20 | 94->104 | 0.59042 | 7.0197 | 176.62 | 0.2789 | -5.0349 | -5.3196 |
|  | 95->104 | -0.25884 |  |  |  |  |  |
| 21 | 93->104 | -0.25747 | 7.1096 | 174.39 | 0.0287 | -10.2682 | -10.4524 |
|  | 94->104 | -0.22681 |  |  |  |  |  |
|  | 101->108 | 0.30601 |  |  |  |  |  |
| 22 | 102->111 | 0.43291 | 7.1485 | 173.44 | 0.0192 | 17.0935 | 19.7664 |
| 23 | 101->107 | 0.36465 | 7.1772 | 172.75 | 0.1179 | 1.8759 | 4.7695 |
|  | 101->108 | 0.2422 |  |  |  |  |  |
| 24 | 90->103 | 0.4795 | 7.2137 | 171.87 | 0.0004 | 2.8889 | 2.762 |
|  | 95->103 | 0.28371 |  |  |  |  |  |
| 25 | 101->107 | -0.26251 | 7.2386 | 171.28 | 0.1943 | 3.3176 | -0.4195 |
|  | 101->108 | 0.3318 |  |  |  |  |  |
|  | 101->109 | 0.23651 |  |  |  |  |  |
| 26 | 102->107 | 0.53444 | 7.2527 | 170.95 | 0.0084 | 6.1761 | 6.1745 |
|  | 102->115 | 0.24468 |  |  |  |  |  |
| 27 | 90->103 | -0.3344 | 7.2977 | 169.89 | 0.0003 | 1.2767 | 1.6045 |
|  | 95->103 | 0.44498 |  |  |  |  |  |
|  | 96->103 | 0.29088 |  |  |  |  |  |
| 28 | 86->103 | 0.35304 | 7.3804 | 167.99 | 0.0011 | -3.3244 | -3.164 |
|  | 89->103 | 0.37377 |  |  |  |  |  |
| 29 | 102->106 | 0.69544 | 7.4063 | 167.40 | 0.0008 | 1.151 | 1.2584 |
| 30 | 102->111 | 0.28476 | 7.4297 | 166.88 | 0.0106 | -5.9703 | -6.7308 |
|  | 102->112 | 0.31077 |  |  |  |  |  |
|  | 102->114 | 0.29182 |  |  |  |  |  |
|  | 102->115 | 0.31729 |  |  |  |  |  |

*^a^*Number of the excited states; *^b^*Only transitions with contribution over 10.0% were listed; *^c^*Configuration-interaction coefficient; *^d^*Excitation energy; *^e^*Wavelength; *^f^*Oscillator strength; *^g^*Rotatory strength in velocity form (10^-40^ cgs); *^h^*Rotatory strength in length form (10^-40^ cgs).

**Table S3.** Key transitions, oscillator strengths, and rotatory strengths in the ECD spectrum of conformer **5-2** at the Cam-B3LYP/TZVP level of theory in MeOH with IEFPCM solvent model.

| ***Num^a^*** | ***Transition^b^*** | ***CI-coeff^b^*** | ***ΔE (eV)^d^*** | ***λ (nm)^e^*** | ***f^f^*** | ***R_vel_^g^*** | ***R_len_^h^*** |
| --- | --- | --- | --- | --- | --- | --- | --- |
| 1 | 102->103 | 0.69613 | 3.6730 | 337.55 | 1.0806 | 126.914 | 128.1276 |
| 2 | 99->103 | 0.62221 | 3.9066 | 317.37 | 0.0060 | -38.3615 | -39.3461 |
|  | 99->105 | 0.26871 |  |  |  |  |  |
| 3 | 101->104 | 0.68194 | 4.2052 | 294.83 | 0.1032 | -50.3638 | -51.9794 |
| 4 | 100->104 | 0.67016 | 5.0459 | 245.71 | 0.4715 | -10.0064 | -10.3637 |
| 5 | 101->103 | 0.69944 | 5.1370 | 241.35 | 0.0006 | -0.9045 | -0.8641 |
| 6 | 102->104 | 0.69462 | 5.4157 | 228.93 | 0.0053 | -1.723 | -1.9498 |
| 7 | 97->103 | -0.23695 | 5.4652 | 226.86 | 0.1577 | 17.4813 | 17.7725 |
|  | 98->103 | 0.62229 |  |  |  |  |  |
| 8 | 95->104 | 0.48351 | 5.7274 | 216.47 | 0.0001 | 0.1139 | 0.3659 |
|  | 96->104 | 0.37933 |  |  |  |  |  |
| 9 | 100->103 | 0.65526 | 5.8054 | 213.57 | 0.0018 | -2.5926 | -2.6011 |
| 10 | 97->103 | 0.59302 | 5.8426 | 212.21 | 0.0122 | -1.7704 | -1.8986 |
|  | 98->103 | 0.24421 |  |  |  |  |  |
| 11 | 102->105 | 0.65978 | 5.9233 | 209.32 | 0.0896 | -6.5967 | -5.2613 |
| 12 | 101->106 | 0.66296 | 6.0814 | 203.88 | 0.5848 | 21.0562 | 21.7122 |
| 13 | 95->103 | -0.29062 | 6.1112 | 202.88 | 0.0086 | -16.4248 | -17.8153 |
|  | 96->103 | 0.49928 |  |  |  |  |  |
| 14 | 92->103 | 0.47785 | 6.3347 | 195.72 | 0.0037 | 5.8462 | 5.8376 |
|  | 93->103 | 0.45251 |  |  |  |  |  |
| 15 | 100->106 | 0.5939 | 6.4242 | 193.00 | 0.1039 | -0.8844 | -0.979 |
|  | 101->108 | 0.28502 |  |  |  |  |  |
| 16 | 93->103 | -0.23488 | 6.6041 | 187.74 | 0.0074 | -15.1928 | -15.6925 |
|  | 99->104 | 0.5486 |  |  |  |  |  |
| 17 | 92->103 | -0.30612 | 6.6550 | 186.30 | 0.0038 | -3.4997 | -3.2498 |
|  | 93->103 | 0.35225 |  |  |  |  |  |
|  | 99->104 | 0.4015 |  |  |  |  |  |
| 18 | 99->103 | -0.25624 | 6.8439 | 181.16 | 0.0017 | 9.2576 | 9.2162 |
|  | 99->105 | 0.45114 |  |  |  |  |  |
| 19 | 102->107 | -0.27355 | 6.9100 | 179.43 | 0.0021 | -9.353 | -8.0258 |
|  | 102->108 | -0.28356 |  |  |  |  |  |
|  | 102->109 | 0.42006 |  |  |  |  |  |
|  | 102->113 | 0.28945 |  |  |  |  |  |
| 20 | 94->104 | 0.587 | 7.0173 | 176.68 | 0.2754 | -6.7462 | -7.6005 |
|  | 95->104 | -0.25239 |  |  |  |  |  |
| 21 | 93->104 | 0.27191 | 7.1152 | 174.25 | 0.0255 | -4.0397 | -6.0645 |
|  | 94->104 | 0.25355 |  |  |  |  |  |
|  | 96->104 | -0.23087 |  |  |  |  |  |
|  | 101->108 | -0.27017 |  |  |  |  |  |
| 22 | 102->111 | 0.42315 | 7.1490 | 173.43 | 0.0129 | 12.4461 | 14.4024 |
| 23 | 101->107 | 0.46859 | 7.2010 | 172.18 | 0.0057 | 0.2124 | 1.0801 |
|  | 101->110 | 0.29676 |  |  |  |  |  |
| 24 | 90->103 | 0.38084 | 7.2115 | 171.93 | 0.1135 | 22.7853 | 26.4659 |
|  | 95->103 | 0.23988 |  |  |  |  |  |
|  | 101->108 | 0.263 |  |  |  |  |  |
| 25 | 90->103 | -0.29606 | 7.2177 | 171.78 | 0.2099 | -17.9259 | -23.1287 |
|  | 100->106 | -0.22435 |  |  |  |  |  |
|  | 101->108 | 0.34535 |  |  |  |  |  |
| 26 | 102->107 | 0.5398 | 7.2530 | 170.94 | 0.0068 | 8.9991 | 8.8135 |
|  | 102->115 | 0.24663 |  |  |  |  |  |
| 27 | 90->103 | -0.33579 | 7.2973 | 169.90 | 0.0002 | 1.1915 | 1.4973 |
|  | 95->103 | 0.44658 |  |  |  |  |  |
|  | 96->103 | 0.29083 |  |  |  |  |  |
| 28 | 86->103 | 0.3533 | 7.3803 | 167.99 | 0.0010 | -3.507 | -3.3217 |
|  | 89->103 | 0.37394 |  |  |  |  |  |
| 29 | 102->106 | 0.69724 | 7.4070 | 167.39 | 0.0007 | 1.0331 | 1.1414 |
| 30 | 102->111 | 0.28244 | 7.4296 | 166.88 | 0.0107 | -5.8912 | -6.6335 |
|  | 102->112 | 0.31276 |  |  |  |  |  |
|  | 102->114 | 0.28998 |  |  |  |  |  |
|  | 102->115 | 0.32222 |  |  |  |  |  |

*^a^*Number of the excited states; *^b^*Only transitions with contribution over 10.0% were listed; *^c^*Configuration-interaction coefficient; *^d^*Excitation energy; *^e^*Wavelength; *^f^*Oscillator strength; *^g^*Rotatory strength in velocity form (10^-40^ cgs); *^h^*Rotatory strength in length form (10^-40^ cgs).

**Figure S1.** ^1^H NMR (500 MHz, DMSO-*d*_6_) spectrum of compound **1**

**Figure S2.** ^13^C NMR (125 MHz, DMSO-*d*_6_) spectrum of compound **1**

**Figure S3.** HSQC spectrum of compound **1**

**Figure S4.** COSY spectrum of compound **1**

**Figure S5.** HMBC spectrum of compound **1**

**Figure S6.** HRESIMS spectrum of compound **1**

**Figure S7.** ^1^H NMR (500 MHz, DMSO-*d*_6_) spectrum of compound **2**

**Figure S8.** ^13^C NMR (125 MHz, DMSO-*d*_6_) spectrum of compound **2**

**Figure S9.** HSQC spectrum of compound **2**

**Figure S10.** COSY spectrum of compound **2**

**Figure S11.** HMBC spectrum of compound **2**

**Figure S12.** HRESIMS spectrum of compound **2**

**Figure S13.** ^1^H NMR (500 MHz, DMSO-*d*_6_) spectrum of compound **5**

**Figure S14.** ^13^C NMR (125 MHz, DMSO-*d*_6_) spectrum of compound **5**

**Figure S15.** HSQC spectrum of compound **5**

**Figure S16.** COSY spectrum of compound **5**

**Figure S17.** HMBC spectrum of compound **5**


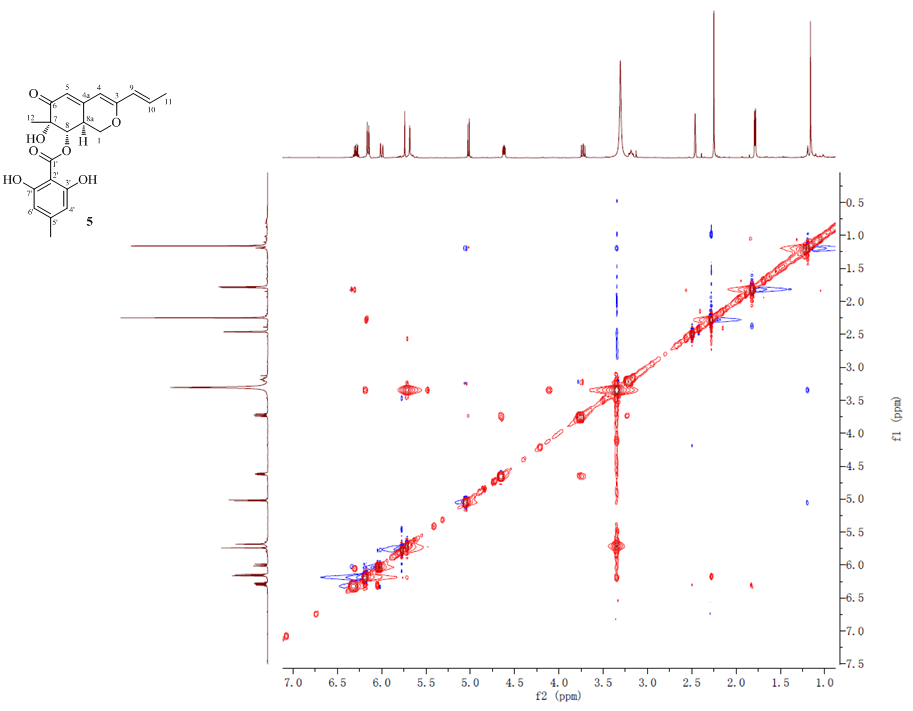


**Figure S18.** NOESY spectrum of compound **5**

**Figure S19.** HRESIMS spectrum of compound **5**

**Figure S20.** Chiral HPLC analysis of compound **5** (Whelk-O1 rpirkle-cencept chiral HPLC column, *n*-hexane−ethanol 10:1, 1.0 mL/min)
